# Roles of Cullin-RING Ubiquitin Ligases in Cardiovascular Diseases

**DOI:** 10.3390/biom12030416

**Published:** 2022-03-08

**Authors:** Stephanie Diaz, Kankan Wang, Benita Sjögren, Xing Liu

**Affiliations:** 1 Department of Biochemistry, Purdue University, West Lafayette, IN 47907, USA; diaz175@purdue.edu (S.D.); wang4196@purdue.edu (K.W.); 2 Department of Medicinal Chemistry and Molecular Pharmacology, Purdue University, West Lafayette, IN 47907, USA; jsjogren@purdue.edu

**Keywords:** ubiquitin E3 ligases, cullin-RING ligases, protein ubiquitination, protein degradation, cardiovascular disease

## Abstract

Maintenance of protein homeostasis is crucial for virtually every aspect of eukaryotic biology. The ubiquitin-proteasome system (UPS) represents a highly regulated quality control machinery that protects cells from a variety of stress conditions as well as toxic proteins. A large body of evidence has shown that UPS dysfunction contributes to the pathogenesis of cardiovascular diseases. This review highlights the latest findings regarding the physiological and pathological roles of cullin-RING ubiquitin ligases (CRLs), an essential player in the UPS, in the cardiovascular system. To inspire potential therapeutic invention, factors regulating CRL activities are also discussed.

## 1. Introduction

For many years, systematic cohort and mechanistic studies have been carried out to unveil risk factors and therapeutic targets for cardiovascular diseases. Consequently, proper management of identified risk factors and application of cognate therapeutic strategies have led to a remarkable decrease in the incidence and fatality of cardiovascular diseases. Despite these efforts, cardiovascular diseases remain a leading cause of mortality around the globe, with 18.6 million recorded deaths in 2019 [1]. Furthermore, as a result of the world population aging, the number of people dying from cardiovascular diseases has been increasing over the last decade, especially in low- and middle-income countries [2]. It is thus still urgent to explore new avenues for the prevention and treatment of cardiovascular diseases.

**Figure 1:**
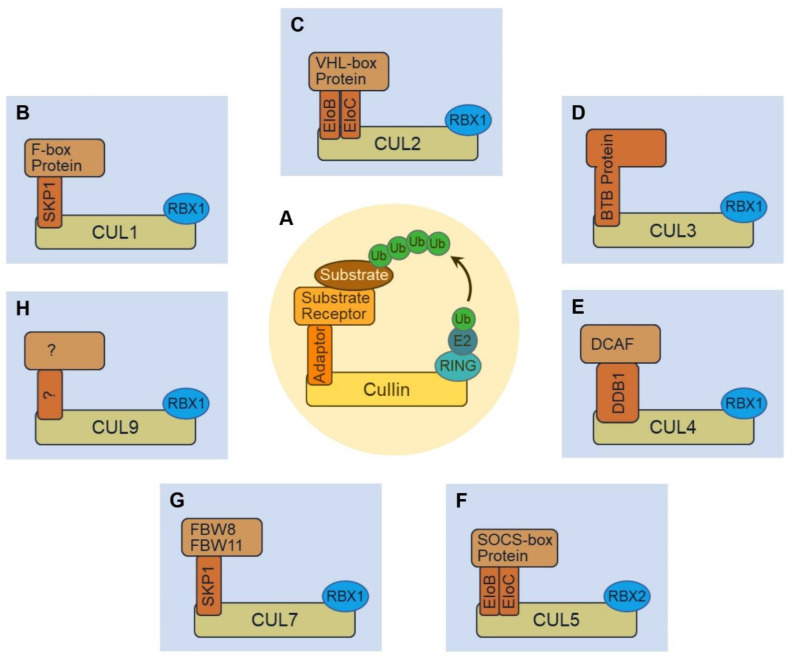
Schematic illustrating the structure of cullin-RING ubiquitin ligases (CRLs). (**A**) CRL complexes are modular. They all contain a cullin protein that serves as the backbone for the complex. A RING finger protein binds the C-terminus of cullin and serves as an adaptor for E2~Ub. The N-terminus of cullin recruits various substrate receptors—usually through an adaptor protein—to recognize and specify target substrates for ubiquitination. (**B**) The CUL1•RBX1 enzymatic core of CRL1 uses SKP1 as an adaptor to recruit F-box proteins as substrate receptors. (**C**) CRL2 use EloB (elongin B) and EloC (elongin C) complex as the adaptor to recruit VHL-box proteins as substrate receptors. (**D**) CRL3 directly recruits BTB proteins without the need of an adaptor protein. (**E**) CRL4 (including CRL4A and CRL4B) uses DDB1 as the adaptor to recruit DCAF proteins as substrate receptors. (**F**) CRL5 is composed of CUL5 and RBX2, and it uses EloB and EloC complex as the adaptor to recruit SOCS-box proteins as substrate receptors. (**G**) CRL7 has been known to use the SKP1 adaptor protein to recruit FBW8 or FBW11 F-box protein substrate receptors. (**H**) CRL9 comprises CUL9 and RBX1, while the adaptor and substrate receptor complex remain unknown.

Under various stress conditions, cells need to properly maintain a functional proteome for fundamental biological processes [3]. The ubiquitin-proteasome system (UPS) represents a pivotal player in regulating protein homeostasis. It has been well-established that UPS-mediated proteolysis is capable of targeting individual proteins for proteasomal degradation [4]. The signal that triggers such degradation is ubiquitination, a post-translational modification where a chain of the small protein ubiquitin (Ub) is covalently linked to a lysine residue of the target protein. Ubiquitination is catalyzed by sequential enzymatic reactions involving the Ub activating enzyme (E1), the Ub conjugating enzyme (E2) and the Ub ligase (E3). The substrate specificity of the ubiquitination system is conferred by E3s, which mediate the transfer of Ub to the target protein. Hundreds of E3s are encoded by the human genome, of which about 40% are predicted to cooperate with the UPS [5]. Based on typical structural characterizations, E3 ligases are classified into three main classes: the really interesting new gene (RING) type, the homologous to E6-AP carboxyl terminus (HECT) type and the RING-between-RING (RBR) type. With more than two hundred members, the multi-unit cullin-RING ligases (CRLs) represent the largest and most studied subfamily of E3 ligases [6,7]. The modular CRL complexes are characterized by a common cullin (CUL) scaffold, a small RING protein (RBX1 or RBX2), and an interchangeable substrate receptor module. Structurally, the C-terminus of the elongated CUL scaffold binds the RBX protein that recruits the E2 conjugated Ub (E2~Ub), whereas the N-terminus of CUL binds the various substrate receptor modules [8]. Seven different types of CUL proteins have been identified in human cells (CUL1, CUL2, CUL3, CUL4A, CUL4B, CUL5, CUL7 and CUL9), each of which engages a family of substrate receptors to assemble active CRLs (Figure 1). In addition, CUL6 has been identified in *C. elegans* [6], and Cul8 has been found in *S. cerevisiae* [9,10], but neither have been reported in humans. Through bringing the substrate and E2~Ub in close proximity, CRLs facilitate substrate ubiquitination [11].

Growing evidence implicates that cardiovascular UPS plays a key role in regulating diverse cellular events such as the function of cardiac membrane channel and receptor, apoptosis of cardiomyocyte and vascular smooth muscle cells, as well as the quality control of sarcomere [12,13,14]. Given the importance of CRLs in the UPS, herein, we review the recent understanding on the role of CRLs in the context of cardiovascular diseases. 

## 2. The CUL1-RING Ligase (CRL1)

The prototype of the CRL family is the CUL1-based CRL1 (also known as SCF) complex, which consists of the CUL1 core, the adaptor protein SKP1 (S-phase kinase-associated protein 1), and an F-box protein substrate receptor (Figure 1B) [8,15]. The RING protein RBX1 binds the C-terminal domain of CUL1 and serves as the docking site for E2~Ub. The N-terminal domain of CUL1 binds SKP1, and SKP1 recruits F-box proteins through their conserved F-box motif. Up to 69 different F-box proteins can be made in human cells [16], each of which recognizes specific protein targets for ubiquitination. Through engaging different F-box protein substrate receptors, CRL1s can change their substrate specificity, and therefore, various proteins can be targeted for ubiquitination and degradation [17,18]. While a multitude of studies have suggested F-box proteins as promising cancer drug targets [19], there is emerging evidence suggesting that therapeutic targeting of F-box proteins could be beneficial in other pathologies as well.

### 2.1. Implications for CRL1^FBXO32^ in Cardiac Function

An F-box protein well-known for its involvement in cardiovascular disease is FBXO32 (or Atrogin-1), a protein specifically expressed in skeletal and cardiac muscles [20]. FBXO32 was firstly identified as a key regulator for skeletal muscle atrophy [21,22,23,24], and it targets the initiation factor eIF3-f [25] and the myogenic regulatory factor MyoD [26] for ubiquitination and degradation. Besides a critical role in skeletal muscle, FBXO32 was also found to enhance ischemia/reperfusion-induced apoptosis in cardiomyocytes through promoting the proteosome-dependent degradation of MAPK phosphatase-1 [27]. In addition, studies using mouse models showed that Fbxo32 regulates the ubiquitination of Foxo1/3 [28], and it is involved in cardiac hypertrophy and cardiac aging [28,29,30,31]. More recently, human mutations in FBXO32 were identified to cause dilated cardiomyopathy [32,33,34], through dysregulating autophagy [32] and upregulating CHOP (C/EBP homologous protein)-mediated apoptosis [34]. Taken together, FBXO32 is a key player in cardiac physiology and the pathogenesis of heart disease.

### 2.2. CRL1^FBXL2^ and Ca^2+^ Homeostasis

A recent study highlighted the role of another F-box protein, FBXL2, in obesity-related cardiac dysfunction [35]. FBXL2 is widely expressed in various human tissues [36,37], and it was first found to bind and ubiquitinate the unphosphorylated p85β subunit of PI(3)Ks (phosphatidylinositol-3-OH kinases) [38]. Defects in the FBXL2-mediated degradation of p85β could promote autophagy [38]. Further searches for the substrate of CRL1^FBXL2^ identified IP3R3, a receptor for IP3 (inositol 1,4,5-trisphosphate), as a direct target of FBXL2 [39]. The FBXL2-dependent degradation of IP3R3 can limit Ca^2+^ influx into mitochondria and reduce apoptosis. In addition, the tumor suppressor PTEN (phosphatase and tensin homologue) was found to compete with FBXL2 for IP3R3 binding, and as a result, PTEN can limit tumor growth through stabilizing IP3R3 and enhancing Ca^2+^-dependent apoptosis [39]. The abovementioned study by Ren et al. [35] found that FBXL2 also interacts with FUNDC1 (FUN14 domain containing 1), an integral mitochondrial outer-membrane protein, in mouse heart. This interaction changes the stability of FBXL2 and, consequently, the stability of IP3R3. Suppression of FUNDC1 activity, which can be triggered by high-fat diet, reduces FBXL2-dependent degradation of IP3R3, leading to mitochondrial Ca^2+^ overload and cardiomyocyte dysfunction [35]. While the mechanism by which FUNDC1 regulates the stability of FBXL2 remains to be fully elucidated, these studies suggest that through altering the activity of CRL1^FBXL2^, it is possible to enhance or suppress Ca^2+^-dependent apoptosis. Therefore, FBXL2 or regulators of CRL1^FBXL2^ can be targets for therapeutic development. 

### 2.3. A Role for CRL1^FBXW7^ in Oxidative Stress

FBXW7 is an F-box protein that has been heavily studied as a tumor suppressor. It is frequently mutated in human cancers, and targets multiple oncoproteins for proteasome-dependent degradation (for review, see [40]). Among these oncoproteins, MCL-1 (myeloid cell leukaemia-1) is an apoptosis regulator that regulates the apoptosis of not only cancer cells but also cardiac cells [40,41,42]. Given the important role of MCL-1 in mitochondria and cardiomyocytes [41], it is unsurprising that FBXW7 is involved in oxidative stress-induced cardiac cell injury [43]. Further, FBXW7 has been reported as a regulator of cardiac hypertrophy, likely through controlling the protein stability of EZH2 (enhancer of zeste homology 2) and the transcription of SIX1 (sine oculis homeobox homolog 1) [44]. Besides regulating the degradation of downstream targets, the expression of FBXW7, in turn, can be regulated by various microRNAs (for review, see [40]), a machinery also implicated in the pathogenesis of cardiovascular disease [45,46]. In fact, FBXW7 is not the only F-box protein known to be regulated by microRNAs. The microRNA MiR-184 was reported to target FBXO28, and inhibiting MiR-184 could reduce H_2_O_2_-induced cardiomyocyte injury [47]. This finding suggests that FBXO28 may play a role in handling oxidative stress and apoptosis in cardiomyocytes. 

### 2.4. CRL1^SKP2^ in Proliferation and Senescence

Another recent study suggests that the F-box protein SKP2 (S-phase kinase-associated protein 2) plays a role in the aging of endothelial progenitor cells [48]. CRL1^SKP2^ has been extensively studied, and through targeting a variety of cell cycle proteins for ubiquitination, SKP2 plays key roles in cell proliferation and tumorigenesis (for review, see [49]). Consistent with its general cellular function, SKP2 regulates the proliferation of vascular smooth muscle cells [50]. Through analyzing the senescence of human endothelial progenitor cells, Wang et al. [48] found that decreased SKP2 levels induce early senescence while increasing the level of SKP2 can partially reverse senescence in aged endothelial progenitor cells. This finding suggests that aging-related vascular disease may be managed by altering the level or activity of CRL1^SKP2^. 

## 3. The CUL2-RING Ligase (CRL2)

### 3.1. CRL2^VHL^-HIF1α Regulatory Axis and Cardiovascular Diseases

CUL2 is a type of cullin that only exists in in multi-cellular organisms [51]. CRL2 employs CUL2•RBX1 as the catalytic scaffold and the Elongin B and Elongin C (EloB/C) protein complex as the adaptor for substrate receptors (Figure 1C) [52,53]. Existing connections between CRL2 and cardiovascular disease are mostly attributed to HIF1α (hypoxia-inducible factor 1α), a well-studied oxygen-responsive substrate of CRL2 [54]. CRL2-dependent degradation of HIF1α requires post-translational hydroxylation of proline residues located in its ODD (oxygen-dependent degradation) domain [55]. Once hydroxylated, HIF1α binds to VHL (von Hippel-Lindau) tumor suppressor protein, a CRL2 substrate receptor, making it a target for CRL2-mediated proteasomal degradation. The activities of prolyl hydroxylase domain (PHD) enzymes that hydroxylate HIF1α are oxygen-dependent [56], and therefore, in hypoxia, HIF1α does not bind to VHL for degradation and is stabilized. The stabilized HIF1α then translocates into the nucleus and binds HIF1β [57]. The resulting HIF1α/β complex functions as a transcription factor that recognizes and binds specific hypoxia response elements (HREs) on the target genes [58]. It has been well-established that the HIF transcriptional complex mediates a broad spectrum of cellular and physiological pathways demanding adaption to hypoxia [59]. 

HIF1α is closely related to cardiovascular diseases in multiple dimensions. For example, in response to ischemia-induced hypoxia, the HIF transcription factor complex activates protective metabolic adaptions, including repression in oxygen-consuming processes and elevation in oxygen-sparing pathways [60]. Furthermore, HIF transcription factors are able to promote angiogenesis by regulating expression of angiogenic factors [61]. A recent review has summarized the role of HIF-1 in cardiovascular diseases in detail [62]. 

### 3.2. CRL2^VHL^ and PROTACs

In addition to regulating the ubiquitination of natural substrates, VHL also plays a key role in drug discovery via the PROteolysis TArgeting Chimera (PROTAC) strategy [63,64,65,66]. PROTAC molecules contain a chemical linker that combines two types of ligands: one binds to VHL, and the other binds a protein that is not a natural substrate of VHL. Therefore, PROTAC can recruit a protein of interest to CRL2^VHL^ and induce its ubiquitination and degradation. Over the past few years, a large number of VHL-based PROTACs have been reported [67,68,69,70,71,72,73,74,75,76,77,78,79,80,81,82,83,84,85] and new PROTACs are actively being developed. While PROTACs, as the majority of other therapeutic strategies directed towards the UPS, are mostly studied for cancer treatment [63], their effects—either positive or negative—on the cardiovascular system are worth further investigation. 

## 4. The CUL3-RING Ligase (CRL3)

Unlike CUL1 or CUL2 that use adaptor protein(s) to engage substrate receptors, the N-terminus of CUL3 binds substrate receptors directly via their BTB (Bric-a-Brac/Tramtrack/Broad) domain (Figure 1D) [8]. The BTB domain also dimerizes, and as a result, CRL3 complexes exist as homodimers [86,87]. Approximately 180 BTB proteins are encoded in the human genome, and about 50 of them have been confirmed as substrate receptors that can potentially recruit protein substrates to CRL3 for ubiquitination [88]. 

### 4.1. CUL3 Mutations and Cardiovascular Diseases

CUL3 has been shown to play a significant role in the cardiovascular system, particularly in regulating blood pressure [89,90,91,92,93,94,95,96,97,98]. Tissue-specific deletion of the *cul3* gene in mouse skeletal muscle cells or cardiomyocytes led to neonatal lethality, because of skeletal muscle dysfunction or severe cardiomyopathy [91]. Deleting *cul3* specifically in mouse smooth muscle cells resulted in impaired vascular function and hypertension [92]. Knocking down *CUL3*, but not other cullin genes (*CUL1*, *CUL2*, *CUL4a*, *CUL5*), in human umbilical vein endothelial cells caused a significantly reduced level of vascular endothelial (VE)-cadherin protein, suggesting that CUL3 plays a critical role in VE-cadherin-mediated endothelial barrier function [93]. What highlights the role of CUL3 in cardiovascular disease is the identification of dominant-negative *CUL3* mutations in patients with severe forms of familial hyperkalemic hypertension (FHHt) [94,95]. These mutations led to impaired splicing and skipping of exon 9 that encodes amino acid residues 403–459, resulting in a truncated form of CUL3 (CUL3^∆403–459^ or CUL3∆9). The CUL3∆9 mutant can still bind RBX1 and BTB substrate receptor to form a CRL3 complex. However, the RBX1•CUL3∆9•BTB protein complex can no longer ubiquitinate its substrate. Instead, CUL3∆9 led to increased autoubiquitination and increased degradation of the substrate receptor protein [96]. In mouse models, CUL3∆9 induced severe hypertension when expressed ubiquitously [97] or in vascular smooth muscle cells [97,98]. 

### 4.2. CRL3^KLHL3^-WNK Regulatory Axis

Studies of FHHt patients revealed mutations in not only CUL3, but also KLHL3 (Kelch-like 3), WNK1 (with-no-lysine kinase 1) and WNK4 [95,99,100,101]. WNK1 and WNK4 are kinases that regulate blood pressure through a signaling cascade that eventually phosphorylates and activates NCC (Na^+^-Cl^−^ cotransporter) and NKCC2 (Na^+^-K^+^-2Cl^−^ co-transporter) [102]. KLHL3 is a BTB-domain-containing protein that binds CUL3 and recruits the WNK proteins for ubiquitination and degradation [103]. Thus, through controlling the turnover rate of WNK proteins, CRL3^KLHL3^ regulates blood pressure [103]. The interaction between KLHL3 and WNK4 was found to depend on the phosphorylation status of KLHL3: phosphorylation at Serine 433 prevented KLHL3 from binding to WNK4 [104]. Mutations in KLHL3 or WNK4 discovered in FHHt patients disrupt the interaction between KLHL3 and WNK4, leading to insufficient degradation of WNK4 and, ultimately, hypertension [105,106,107,108]. In addition, KLHL2, a homologue of KLHL3, was also reported to form a CRL3 that targets WNK4 for ubiquitination and degradation, so KLHL2 may also play a role in the pathogenesis of FHHt [109].

### 4.3. CRL3^KEAP1^-NRF2 Regulatory Axis

KEAP1 is one of the best-studied substrate receptors for CUL3 [8], and the CRL3^KEAP1^ complex targets NRF2 (nuclear factor-E2-related factor 2) for ubiquitination and degradation. The KEAP1-NRF2 system provides an important mechanism for defense against oxidative and electrophilic stress (for review, see [110]). NRF2 is a transcription factor that activates the transcription of antioxidant genes. Under normal conditions, KEAP1 binds newly synthesized NRF2 in the cytoplasm and keeps it constitutively ubiquitinated and degraded. When exposed to oxidative stress, the cysteine residues of KEAP1 are modified by reactive oxygen species, releasing NRF2 from CRL3^KEAP1^. NRF2 is then stabilized and translocates into nuclei to activate antioxidant defense responses [110]. This mechanism for NRF2 activation is important for cellular redox homeostasis in general and has been observed as a mechanism for cardiovascular protection [111,112,113,114,115,116]. Particularly, NRF2 plays a protective role in cardiomyocytes after myocardial ischemia and reperfusion injury [112,117,118], and in endothelial injuries induced by oxidative stress [119]. Dysregulation of NRF2 may result in chronic heart failure [120] or hypertension [121].

### 4.4. Other BTB Proteins Involved in Cardiovascular Diseases

A few additional BTB proteins have also been reported to contribute to cardiovascular health. First, RhoBTB1 (Rho-related BTB domain-containing 1) is the substrate receptor mediating the CUL3-dependent ubiquitination of PDE5 (phosphodiesterase 5), which is a key regulator for smooth muscle relaxation [122,123]. Consequently, the RhoBTB–PDE5 system regulates vascular smooth muscle function and can protect against hypertension [122]. Second, LZTR1 (leucine zipper-like transcription regulator 1) can bind CHMP1B (charged multivesicular protein 1B) and control the ubiquitination of CHMP1B in a cullin-dependent manner [124]. Mutations of LZTR1 were identified in Noonan syndrome patients with bleeding disorders, and these mutations led to defects in LZTR1-mediated ubiquitination of CHMP1B and, ultimately, impaired vesicle trafficking, resulting in cardiovascular dysfunction [124].

Rho-GTPases are key regulators of cytoskeleton dynamics and cell adhesion [125], and this mechanism also controls the function and activity of endothelial cells [126]. RhoA, a member of the Rho-GTPase family, was found to be recruited to CUL3 for ubiquitination through BTB proteins named BACURDs [127], and the CRL3^BACURD1^-mediated RhoA ubiquitination was impaired by the dominant-negative mutant CUL3∆9 [128]. Another BTB protein, KCTD10 (potassium channel tetramerization domain containing 10), was reported to target RhoB for ubiquitination [126]. The ubiquitinated RhoB was subsequently degraded by lysosomes in endothelial cells, which maintained the integrity of the endothelial barrier [126]. Moreover, mutations in KLHL24 (Kelch like family member 24) were found to cause hypertrophic cardiomyopathy, a common inherited cardiovascular disorder, and silencing the *klhl24a* gene in zebrafish caused defects in cardiac function [129]. These findings revealed a crucial role of KLHL24 in heart development and function, and it will be important to identify the specific protein targets that KLHL24 recognizes for ubiquitination in cardiomyocytes. In summary, through targeting diverse protein substrates for ubiquitination, the CRL3 ubiquitin ligases play key regulatory roles in the cardiovascular system from different perspectives. 

## 5. The CUL4-RING Ligase (CRL4)

Two members of the CUL4 subfamily exist in humans, CUL4A and CUL4B. They share highly similar amino acid sequences, except that CUL4B contains an elongated N-terminal domain of ~150 amino acid residues [130,131]. Both forms of CUL4 use a large protein DDB1 (DNA damage protein 1) as the adaptor to dock DCAF (DDB1–CUL4-associated factor) substrate receptor proteins (Figure 1E). Based on sequence analysis, about 100 DCAF proteins are predicted to associate with DDB1 in human cells [132,133]. 

### 5.1. CRL4^DCAF8^ and CRL4^DDB1^-GRK5

While roles of CRL4s in the cardiovascular system remain largely unexplored, Cul4a overexpression in H9c2, a cell line derived from rat heart tissue, was reported to reduce oxidative stress-induced apoptosis, whereas CUL4A knockdown had the reverse effect [134]. When *cul4b* was specifically knocked out in adipocytes, the mutant mice on a high-fat diet accumulated more body fat and were, thus, more likely to develop obesity [135]. In addition, in the mouse myoblast cell line C2C12, DCAF8 was found to bind TRIM63 (tripartite motif containing 63), a muscle-specific RING-finger ubiquitin ligase that facilitates the ubiquitination of MyHC (myosin heavy chain proteins). C2C12 cells lacking DCAF8 were defective in MyHC degradation and were resistant to atrophy [136]. The mechanism by which TRIM63 and CRL4^DCAF8^ controls the degradation of MyHC is still unclear, and if/how the TRIM63•CRL4^DCAF8^ complex contributes to cardiac function warrants further investigation. Lastly, dysregulation of GRKs (G-protein-coupled receptor kinases) can be associated with pathological conditions including cardiovascular disease [137], and GRK5 was found to form a complex with CUL4 through DDB1 and undergo CUL4•DDB1-dependent ubiquitination [138]. Whether the DDB1–GRK5 interaction requires a DCAF remains unclear and needs further investigation. 

### 5.2. Non-Canonical CRL4s Involved in Cardiovascular Diseases

An interesting link between CRL4 and the cardiovascular system comes from discoveries of non-canonical CRL4 complexes. For example, Grk2, another member of the GRK family, is recognized by Gβ (G protein β subunit) protein and recruited to Cul4a through Ddb1 in mice [139]. This CRL4^Gβ^-dependent degradation of Grk2 plays a protective role in the mouse heart [139]. Another example is the CUL4B•DDB1•FBXO44-dependent ubiquitination of RGS2 (regulator of G protein signaling 2) [140], a protein that regulates vasoconstriction and the lack of which leads to hypertension in mice [141]. Several RGS2 mutations, causing reduced expression due to an increased rate of proteasomal degradation, have also been associated with hypertension in humans [142,143,144]. RGS2 was found to be recruited to CUL4B via an F-box protein, FBXO44, using DDB1 as the adaptor protein. Like the other 68 members of the F-box protein family, FBXO44 can also associate with CUL1 ligases. However, FBXO44 is only capable of targeting RGS2 for ubiquitination in the context of CUL4B but not CUL1 [140]. The RGS2–FBXO44 interaction can be regulated by phosphorylation, and the phosphorylation of Ser^3^ on RGS2 could protect RGS2 from degradation through reducing its binding with FBXO44 [145]. Since low RGS2 protein level is associated with disease such as hypertension and heart failure, drugs that interfere with the RGS2–FBXO44 interaction can be beneficial for preventing cardiovascular diseases [145]. 

### 5.3. CRL4 and PROTACs

A couple of CRL4s have been identified as drug targets for small-molecule-induced protein degradation. As a type of PROTACs (see Section 3), when these “molecular glue” drugs bind to the DCAF protein, the DCAF can then bind a disease-causing protein, leading to CRL4-dependent degradation of the target protein. One such example is the Immunomodulatory Drugs (IMiDs), which bind the Cereblon (CRBN) DCAF protein and trigger the degradation of a variety of cellular regulators, including Ikaros, Aiolos, ZFP91 zinc finger protein, etc. [146,147,148,149,150,151,152,153]. The other example is indisulam, which enables CRL4^DCAF15^ to recruit RBM39 (RNA binding motif protein 39) for ubiquitination and degradation [154,155]. While IMiDs represent an exciting new strategy for drug discovery and have been successfully used to treat patients with multiple myeloma, they also appear to increase the risk of cardiotoxicity in multiple myeloma patients [156]. Thus, the side effects of IMiDs and other molecular glue drugs on the cardiovascular system need to be carefully evaluated to guide and improve their clinical use. 

## 6. The CUL5-RING Ligase (CRL5)

CRL5s recruit E2~Ub through RBX2 that associates with the C-terminus of CUL5. Although recombinant CUL5 could bind recombinant RBX1 in vitro, the formation of the CUL5•RBX1 complex in human cells has not been reported [157]. Like CUL2, the N-terminus of CUL5 employs the EloB/C adaptor complex to associate with different substrate receptors (Figure 1F). However, unlike CUL2 that binds a VHL-box in substrate receptor proteins, CUL5 specifically recognizes the SOCS (suppressor of cytokine signaling)-box at the C-terminus of its substrate receptor proteins [158]. In the human genome, 37 SOCS-box substrate receptors have been identified [157]. 

### CRL5^ASB2^ in Cardiac Development

While the importance of CRL5-dependent protein ubiquitination in cancers has been well-documented [157], what role each CRL5 plays in the cardiovascular system has just started to come to light. ASB2 (ankyrin repeat and SOCS box containing 2) is a CRL5 substrate receptor worth highlighting here. The expression of ASB2 was primarily detected in human cardiac and skeletal muscles [37,159], and *Asb2* knockout mice were embryonic lethal due to cardiovascular defects [160]. ASB2 was found to target Filamin A for ubiquitination and degradation [160,161], thereby controlling actin remodeling in immature cardiomyocytes, which plays an essential role in heart development [160]. Furthermore, SMAD9 (SMAD family member 9) was also identified as a substrate for CRL5^ASB2^ [162]. SMAD9 is one of the transcriptional modulators for BMP (bone morphogenetic protein) signaling, and excessive levels of SMAD9 could lead to abnormal cardiac differentiation [162]. Thus, controlling the stability of SMAD9 and its downstream BMP signaling is another pathway by which CRL5^ASB2^ contributes to normal heart development. 

## 7. The CUL7-RING Ligase (CRL7) and Cardiac Signal Transduction

CUL7 belongs to one of the non-canonical cullins, and its molecular weight is at least twice as large as that of the canonical cullins (CUL1-5). Consistent with canonical cullins, CUL7 contains the conserved cullin domain that binds RBX1 to recruit E2~Ub [163]. Similar to CUL1, CUL7 engages substrate receptors via the adaptor protein SKP1, but only FBXW8 and FBXW11 have been reported to form active E3 ligase complexes with CUL7 (Figure 1G) [164,165,166]. 

The CUL7 protein is present in human muscles (cardiac, smooth, and skeletal) [36,37], and CRL7^FBXW8^ has been shown to regulate insulin signaling in human cells and mice through targeting IRS1 (insulin receptor substrate 1) for ubiquitination and degradation [167,168,169]. In addition, knocking down *Cul7* but not *Cul2*, *Cul3* or *Cul5* in cultured neonatal rat ventricular cardiomyocytes (NRVCs) led to reduced ubiquitination and degradation of Mst1, a key component of the Hippo–YAP signaling pathway [170]. Thus, through controlling the stability of Mst1, CRL7 activities are important for heart development. Furthermore, when the *Cul7* gene was deleted specifically in mouse cardiomyocytes, phosphoinositide 3-kinase (PI3K)/AKT signaling was activated in the heart, cardiomyocyte apoptosis was reduced, and cardiac fibrosis following transverse aortic constriction (an experimental model for pressure overload-induced cardiac hypertrophy and heart failure) was attenuated [171]. These findings suggest that CRL7 can be a target for developing antimyocardial fibrosis therapeutics. 

## 8. The CUL9-RING Ligase (CRL9) and Cardiovascular Disease

CUL9 is the other known non-canonical cullin protein whose functions and properties have not been fully characterized yet. Its sequence is highly similar to CUL7, and with over 2500 amino acids, it is the largest member in the cullin family [172]. The C-terminus of CUL9 binds RBX1, and it also contains a RING between the RING domain. To date, no adapter protein for CUL9 has been identified (Figure 1H) [173]. 

CUL9 has been shown to bind and activate p53, and this interaction is important for cell proliferation and genome integrity [174]. A genome-wide association study (GWAS) revealed that *CUL9* is a risk factor for cardiovascular diseases [175], but the mechanistic link between CUL9 and cardiovascular diseases remains to be further explored. 

## 9. Regulators of CRLs

### 9.1. Neural Precursor Cell Expressed, Developmentally Downregulated 8 (NEDD8)

#### 9.1.1. Neddylation Promotes the Activity of CRLs

The activity of CRLs is tightly regulated by neddylation, a post-translational modification where the ubiquitin-like protein NEDD8 is conjugated to a conserved lysine located in the C-terminal domain of cullins [176,177]. Similar to ubiquitination, the process of cullin neddylation requires consecutive enzymatic reactions involving NEDD8 E1 activating enzyme (NAE), NEDD8 E2 conjugating enzyme, and NEDD8 E3 ligase [18]. Neddylation of cullins efficiently promotes the activity of CRLs, leading to increased ubiquitination of substrates [178,179,180,181]. The mechanism by which neddylation activates CRLs has been revealed from both biochemical and structural perspectives. Biochemically, neddylation promotes ubiquitin chain initiation and elongation through enhancing E2 recruitment and E2 activity [182]. Structurally, neddylation induces substantial conformational changes on the C-terminal domain of the CUL•RBX1 core [183] and triggers multiple protein–protein interactions within the CRL complex, including interactions between NEDD8 and E2, and interactions between E2 and the substrate receptor [184]. As a result, neddylation converts the CRL from an open conformation, which benefits the engagement of E2~Ub and diverse substrates, to a closed and compact conformation, which promotes the transfer of the donor ubiquitin to the acceptor lysine and triggers efficient substrate ubiquitination [185]. Furthermore, cullin neddylation enables the ARIH family of RBR E3 ligases to bind CRLs—ARIH1 binds neddylated CUL•RBX1, whereas ARIH2 binds neddylated CUL5•RBX2—and the ARIH E3 ligase accelerates the transfer of Ub from an E2 to the CRL-bound protein substrate [186,187,188].

#### 9.1.2. Impaired Neddylation Results in Cardiomyopathy

Given the large number of CRL substrates that function as key regulators in the cardiovascular system, it is not surprising that neddylation was reported to be crucial for cardiac development. Genetic studies showed that mice lacking NAE exhibit proliferation arrest in cardiomyocytes and ventricular non-compaction, leading to heart failure and, eventually, neonatal lethality [170]. Transient treatment of MLN4924 (or pevonedistat), a small-molecule inhibitor of neddylation [189], also resulted in obvious cardiac abnormalities in neonatal rats such as reduced cardiomyocyte proliferation and cardiac hypertrophy [190], indicating a role of neddylation in perinatal cardiac growth. Mechanistically, neddylation was shown to promote cardiac chamber maturation partially via the CUL7–MST1–YAP axis (see Section 7) [170]. It is noteworthy that dozens of neddylation substrates other than cullin proteins have been reported [191], and how these substrates contribute to the cardiovascular system remains largely unknown.

### 9.2. COP9 Signalosome (CSN)

#### 9.2.1. CSN Is Required for Maintaining the CRL Activities in Cells

The reverse process of cullin neddylation, termed deneddylation, is mediated by the constitutive photomorphogenesis 9 (COP9) signalosome (CSN) [192], an evolutionarily conserved multi-protein complex. The mammalian CSN complex contains eight subunits in which only CSN5 renders the isopeptidase activity required for deneddylation. Early in vitro biochemical studies showed that CSN inhibits the activity of CRL1 [192], which is consistent with its deneddylase activity. However, later genetic studies demonstrated that CSN is required for CRL-mediated substrate degradation [193,194,195], indicating a positive effect on CRL activity. This functional paradox was partially explained by the role of CSN in preventing autoubiquitination of CRL substrate receptors [196,197,198]. More recently, with the functional characterization of CAND1 (cullin-associated NEDD8-dissociated protein 1; see also Section 9.3) [199,200,201], an alternative model was proposed that CSN promotes CRL activities by allowing CAND1 to bind cullins and subsequently exchange the substrate receptor module associated with the CUL•RBX1 core [202] (see Section 9.3). 

#### 9.2.2. Multifaceted Roles of CSN in Cardiac Physiology

CSN has been implicated in the pathogenesis of cardiovascular diseases by various studies [203,204,205,206,207,208,209,210,211,212]. First, cardiac-specific knockout of *Csn8* in mice resulted in defective Csn complex assembly, leading to cardiac hypertrophy, heart failure, and eventually, postnatal lethality four to five weeks after birth [210]. This finding, together with phenotypes observed in NAE-deficient mice [170], highlights the importance of the neddylation–deneddylation cycle in heart development. Moreover, conditional deletion of *Csn8* in adult mice hearts caused striking cardiomyocyte necrosis and heart failure [213], suggesting that CSN also plays an essential role in post-mitotic cardiac physiology. Second, it has been shown that through regulating the degradation of misfolded proteins, CSN protects cardiomyocytes from proteotoxic stress [212]. Third, CSN exhibited anti-atherogenic capacity in endothelial and myeloid cells through negatively regulating inflammatory processes [214,215]. In addition to mediating the deneddylation of cullin proteins, CSN also functions as a docking platform for kinases and deubiquitinases [216]. Therefore, besides being a key regulator for CRL-dependent protein ubiquitination and degradation, CSN may also play a CRL-independent role in the cardiovascular system. 

### 9.3. Cullin-Associated NEDD8-Dissociated Protein 1/2 (CAND1/2)

**Figure 2:**
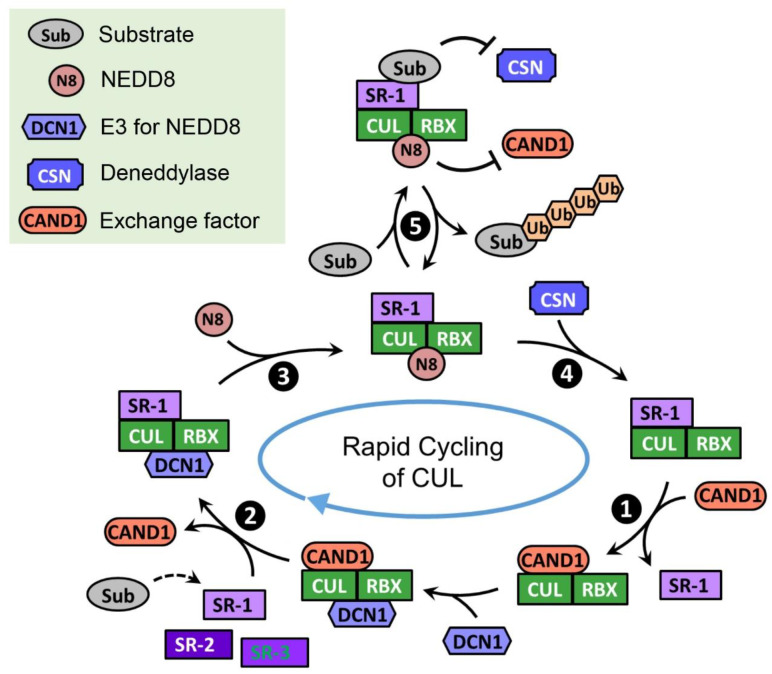
Model for the rapid cycling of CUL. In the absence of CRL substrates, CUL quickly cycles through the CRL assembly (**❷**), neddylation (**❸**), deneddylation (**❹**), and exchange states (**❶**). This rapid cycling of CUL enables all kinds of substrate receptors to access the limited amount of CUL and assemble active CRLs that can potentially ubiquitinate their substrates. When the substrate is loaded on the CRL, it prevents CSN from binding and thus stabilizes CRL to allow substrate ubiquitination (**❺**). Key factors regulating the cycling of CUL are listed in the green box.

CAND1 is a substrate receptor exchange factor for CRLs, and it binds unneddylated cullins in a manner mutually exclusive with substrate receptors [202]. When cullins are bound by CAND1, cullin neddylation is inhibited but the engagement of neddylation enzymes to the cullin is increased [201]. In the absence of CAND1, a CRL1 complex is very stable and displays extremely slow dissociation. CAND1 can dramatically increase the dissociation rate of the CRL1 and binds tightly to the CUL1 “recycled” from the pre-existing CRL1 complex (Figure 2, Step 1). Subsequently, a new substrate receptor module can destabilize the CUL1•CAND1 complex, remove CAND1, and form a new CRL1 (Figure 2, Step 2). This exchange process is controlled by neddylation: immediately after the removal of CAND1 by the substrate receptor module, CUL1 is neddylated and can no longer bind to CAND1 (Figure 2, Step 3); only when NEDD8 is cleaved by CSN, CUL1 is subject to CAND1-mediated exchange (Figure 2, Step 4). Substrate binding inhibits the NEDD8 deconjugation by CSN [217,218], allowing the formation of an active and stable CRL1 that can efficiently ubiquitinate the substrate (Figure 2, Step 5). With the collaborated efforts from CAND1, NEDD8, and CSN, CRL1 complexes constantly undergo cycles of assembly and disassembly, and substrate receptor modules in CRL1 complexes are rapidly exchanged [201,219]. This CAND1-mediated cycling of CUL1 and substrate receptor exchange has been primarily studied in the CRL1 system, but the same mechanism has been shown to apply to CRL3s [220] and CRL4s [221]. The rapid exchange cycles allow cells to quickly adjust the CRL repertoire in response to changing substrate demands and, therefore, newly emerged CRL substrates can be timely ubiquitinated. 

In mammals, CAND1 has a homologue, CAND2. The amino acid sequence of CAND2 is highly similar to CAND1, and CAND2 binds CUL1 in mammalian cells [201,222]. Unlike CAND1 that is ubiquitously expressed in all types of human cells, CAND2 protein is only detected in striated muscles (skeletal and cardiac) and testis [36,37,222]. While the role of CAND2 in regulating CRLs has not been well elucidated yet, population genetics and genome-wide association studies have identified *CAND2* as a risk factor for multiple types of cardiovascular diseases (especially atrial fibrillation) [223,224,225,226,227,228,229]. A recent study aiming to determine the mechanism by which mTOR promotes pathological cardiac remodeling identified *cand2* as a gene significantly upregulated by mTOR in mouse cardiomyocytes, and CAND2 depletion led to decreased protein level of Grk5 in a Cul1-dependent manner [230]. Furthermore, *cand2* knockout mice exhibited pathological remodeling in the heart [230]. These results implied that an increased level of CAND2 would stabilize Grk5 and lead to adverse cardiac remodeling, providing one example for how CAND2 can be involved in cardiovascular diseases. 

## 10. Concluding Remarks

Since the discovery of the F-box motif two and a half decades ago, the field of CRLs has been developing rapidly: components of the CRL family are defined, structures of diverse CRLs are solved, and substrates of individual CRL are discovered. It has now become clear that CRL-mediated ubiquitination modulates the activity and stability of proteins that play crucial roles in human cells, and thereby, CRLs regulate a broad spectrum of biological events. Furthermore, because of their capacity to selectively target cellular proteins for degradation, CRLs provide abundant opportunities for drug discovery, beyond the already clinically used proteasome inhibitors that have been proven successful for the treatment of cancer. The selectivity towards a limited number of substrates targeted by each CRL benefits drug discovery in other pathologies, where the tolerance for severe side effects is much lower, including hypertension and other cardiovascular diseases. In addition, the novel and promising PROTAC technique provides yet another avenue to harness the power of CRLs in the control of protein homeostasis. 

With recent studies in cell biology, physiology, and disease genetics, CRL functions are implicated in various cardiovascular diseases. Continued efforts in understanding the mechanism and regulation of CRLs will undoubtedly expand our knowledge for the pathogenesis of cardiovascular diseases and uncover new avenues to develop therapeutics for disease treatment and prevention.
